# Redeposition-Free Deep Etching in Small KY(WO_4_)_2_ Samples

**DOI:** 10.3390/mi11121033

**Published:** 2020-11-24

**Authors:** Simen Mikalsen Martinussen, Raimond N. Frentrop, Meindert Dijkstra, Sonia Maria Garcia-Blanco

**Affiliations:** Optical Sciences Group, MESA+ Institute for Nanotechnology, University of Twente, P.O. Box 217, 7500 AE Enschede, The Netherlands; r.n.frentrop@utwente.nl (R.N.F.); m.dijkstra@utwente.nl (M.D.); s.m.garciablanco@utwente.nl (S.M.G.-B.)

**Keywords:** KY(WO_4_)_2_, KYW, tungstate, etching, integrated optics, fabrication, redeposition, reticulation, edge bead, hard mask

## Abstract

KY(WO_4_)_2_ is a promising material for on-chip laser sources. Deep etching of small KY(WO_4_)_2_ samples in combination with various thin film deposition techniques is desirable for the manufacturing of such devices. There are, however, several difficulties that need to be overcome before deep etching of KY(WO_4_)_2_ can be realized in small samples in a reproducible manner. In this paper, we address the problems of (i) edge bead formation when using thick resist on small samples, (ii) sample damage during lithography mask touchdown, (iii) resist reticulation during prolonged argon-based inductively coupled plasma reactive ion etching (ICP-RIE), and (iv) redeposited material on the feature sidewalls. We demonstrate the etching of 6.5 µm deep features and the removal of redeposited material using a wet etch procedure. This process will enable the realization of waveguides both in ion-irradiated KY(WO_4_)_2_ as well as thin KY(WO_4_)_2_ membranes transferred onto glass substrate by bonding and subsequent polishing.

## 1. Introduction

Potassium double tungstates like KY(WO_4_)_2_, KLu(WO_4_)_2_, and KGd(WO_4_)_2_ have been in use as laser crystals for decades. They are versatile materials that exhibit excellent optical properties such as a high Raman gain [1] and high-emission and -absorption cross-sections when doped with rare-Earth ions [2]. Laser sources developed in these materials include bulk ultrafast [3,4,5,6], high-power [4,7], and Raman [8,9,10] lasers and high-gain waveguide amplifiers [11,12] and lasers [7,13,14,15,16,17,18,19] in low-index contrast waveguides. However, higher refractive index contrast integrated waveguides are desirable because of their high field confinement, which leads to low laser threshold power, higher laser efficiency, and permits the realization of structures requiring tight bends, such as ring resonator lasers.

High refractive index contrast waveguides have recently been proposed based on a combination of dry and wet etch processes on swift heavy ion-irradiated KY(WO_4_)_2_ slabs [20,21]. These slabs have a buried region where the refractive index is ~1.85 at 1550 nm, corresponding to amorphous KY(WO_4_)_2_, whereas the top crystalline layer has a refractive index around 2 at 1550 nm. The buried layer can be wet-etched using HCl and tetramethylammonium hydroxide (TMAH) [20], which enables the formation of suspended structures. Pedestal microdisks have been demonstrated using this process [20]. However, the dry-etched trench should penetrate the barrier completely in order for the HCl solution to reach it. This requires achieving an etch depth of more than 5 µm [21] to produce the suspended structures.

Deep etching capabilities are also necessary for the fabrication of high refractive index contrast waveguides on KY(WO_4_)_2_ membranes obtained by bonding followed by mechanical lapping and polishing [22,23,24]. Lapping and polishing very thin layers carries great risk of sample damage, while thicker layers are less fragile. Deep etch processing on layers several µm thick may be a viable high yield approach to device fabrication in KY(WO_4_)_2_.

Major problems encountered in the deep etching of KY(WO_4_)_2_ include the presence of a large edge bead, damage to the brittle KY(WO_4_)_2_ sample during the lithography process, and resist reticulation and redeposition of sputtered KY(WO_4_)_2_ material, which affects the quality of the waveguide sidewalls. The formation of an edge bead is a well-known problem in all photolithography processing [25]. However, edge bead formation is exacerbated by the viscous resist needed to achieve deep etching in combination with the typically small size of KY(WO_4_)_2_ substrates. Furthermore, most photolithography equipment available in multiuser cleanrooms is optimized for 4-inch wafers, and mask angle alignment procedures (i.e., wedge compensation) are not guaranteed to work in small brittle samples. This may cause unnecessary damage both to the sample and to the chromium layer of the photomask. For these reasons, it is desirable to design special tools and adapters for small samples.

Deep sputter etching using a photoresist mask also carries the risk of extreme mask reticulation [26], which leads to a rapid increase of the roughness, delamination, and bubble formation in the resist layer. Photolithographically defined features are in this way destroyed before the desired etch depth has been reached. While most photoresists are susceptible to reticulation, this phenomenon can be overcome through hard mask processing.

Finally, sputter etching in KY(WO_4_)_2_ causes redeposition. Many techniques including rounded masks, thin hard masks, chemical etching, and angled etching can be used to avoid or minimize redeposition [27]. However, these techniques often come at the cost of reduced resolution and increased roughness. One method that has not been shown in KY(WO_4_)_2_ before is to remove the redeposition after dry etching of the waveguides, using a wet etch process step. In this paper, we report the development of sample handling tools to eliminate both the edge bead during spinning and the damage to the samples during UV contact lithography. We also describe in detail the implementation of a hard mask process for deep reactive ion etching and a method to strip redeposition. The combination of these developments facilitates the fabrication of high refractive index contrast waveguides in KY(WO_4_)_2_ while increasing the yield of the process.

## 2. Overview of Complete Process Flow

**Figure 1:**
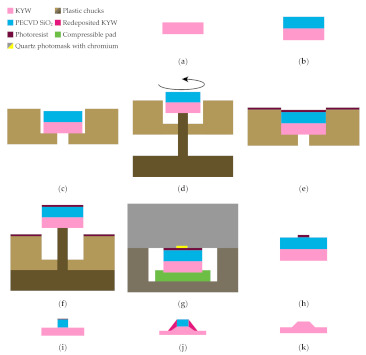
Complete process flow for sample fabrication. (**a**) Initial KY(WO_4_)_2_ sample. (**b**) Plasma-enhanced chemical vapor deposition (PECVD) of SiO_2_ hard mask layer. (**c**) Mounting of sample in spin coating chuck. (**d**) The base tool is used to lift the sample and rotate it into corner contact with the chuck edges. (**e**) Edge bead-reduced spin coating of photoresist. (**f**) Retrieval of sample from the spin coating chuck using the base tool. (**g**) Photolithography exposure using custom mount. (**h**) Development of photoresist. (**i**) Inductively coupled plasma reactive ion etching (ICP-RIE) to open SiO_2_ hard mask. (**j**) Ar-based ICP-RIE etch to pattern KY(WO_4_)_2_. (**k**) Redeposition stripping using 20% HCl at 80 °C and mask removal in HF.

The complete process flow proposed in this work for the fabrication of high refractive index contrast waveguides in KY(WO_4_)_2_ is shown in Figure 1. The main innovations introduced by this work are shown in steps c–g and in k. Steps c–f show our approach to the spin coating process using thick photoresist on small samples, in this case 1 cm^2^, with minimal edge bead. Step g shows a tool used during the exposure step to reduce risk of sample cracking during UV contact lithography. Furthermore, redeposited material from the inductively coupled plasma reactive ion etching (ICP-RIE) process is removed from the sidewalls of the feature using a selective wet etch (Figure 1k). The KY(WO_4_)_2_ samples used in this work are purchased from Altechna, LT. They have a size of 10 mm × 10 mm × 1 mm. In the following sections, the different steps of the described process flow are detailed.

## 3. Edge Bead Removal

Resist edge bead is a well-known problem in spin coating. During spinning, resist forms a thicker ridge on the edge of the wafer [25] called edge bead. The presence of the edge bead can cause flaking, poor photomask contact, photomask contamination in case of insufficient drying, and a reduction of useful processing area in the sample. Edge bead issues are worse when using viscous, thick resists, which is necessary for deep etching. Furthermore, the problem is further enhanced when using small samples, as a large proportion of the sample is close to the edge.

In the processing of full wafers, edge bead removal is typically performed using spray nozzles dispensing solvents on the backside or edge of the wafer. On small, square samples, such a procedure is not an option. While it is possible to remove the edge bead manually using a swab dipped in a solvent, this processing step is not reproducible and introduces additional solvents and potential contamination to the surface of the sample.

**Figure 2:**
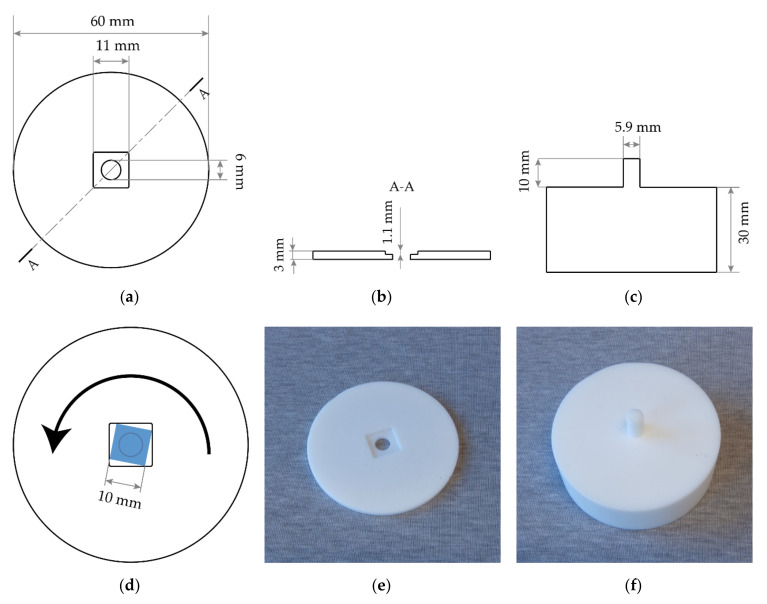
Technical drawing of the chuck used for edge bead-reduced spin coating. (**a**) Top view of the chuck. (**b**) Cross-section through line A-A in (**a**), showing the thickness of the chuck and depth of the recess. (**c**) Cross-section of the base plate. (**d**) Schematic of the chuck with the sample mounted, with the direction of rotation indicated. (**e**–**f**) Photographs of the spin coating chuck (**e**) and sample removal tool (**f**).

In this work, a special spin-coating chuck was designed, shown in Figure 2. The chuck consists of a circular disk 6 cm in diameter, (Figure 2a,b,d,e), which matches an existing chuck that is firmly mounted on the spin coater utilized in this work (i.e., custom-made spin coater in the MESA+ Nanolab). The custom chuck can be placed and removed from the existing chuck in the same way as a regular wafer and is held in place by vacuum. A square blind hole of size 11 mm × 11 mm and 1.1 mm in depth has been milled in the center of the custom chuck, designed to fit the 10 mm × 10 mm × 1 mm KY(WO_4_)_2_ sample. The blind corners have a radius of 400 µm due to the round milling tool. A circular hole of diameter 6 mm was milled in the center of the blind. This hole provides contact with the vacuum lines, which protects the KY(WO_4_)_2_ sample from being disturbed by centrifugal forces.

A base tool was also designed, which consists of a thick base with a 10-mm long protruding pillar with a diameter of 5.9 mm (Figure 2c,f). This tool is used during the mounting and dismounting of the sample.

The sample is mounted in the chuck in a 3-step process, shown in Figure 1c,d: first the sample is placed in the center of the blind with tweezers (Figure 1c). Second, the chuck is lowered slightly onto the base plate, such that the sample is raised without leaving the blind (Figure 1d). The chuck is then rotated counterclockwise, which brings the corners of the sample into contact with the edges of the blind, shown in Figure 2d. It is very important to ensure a good contact of the sample with the spinning chuck so that the photoresist, forced to the corners by centrifugal forces, gets transferred from the sample to the chuck, thereby minimizing edge bead formation. After spinning, the chuck is lowered onto the base plate (Figure 1f) in order to raise the sample, which can then easily be picked up from the tool.

### 3.1. Characterization of the Performance

Four KY(WO_4_)_2_ samples were spin-coated with an Olin OiR 908-35 photoresist at a spin speed of 2000 rpm for 60 s, for a thickness of 5 µm. Before resist spinning, the samples were cleaned for 10 min in 99% HNO_3_, dehydration baked at 120 °C for 5 min, and spin-coated with hexamethyldisilazane (HMDS) at 4000 rpm. First, a regular spin coating chuck (chuck 1) with a small vacuum hole was used. Afterward, the procedure was repeated using the custom spin coating chuck described in this paper (chuck 2).

After spin coating, the samples were soft baked at a temperature of 90 °C for 2 min. Comparison of the samples was performed using optical microscopy. Images were taken of all 4 corners of the samples, and the length of the edge bead along the edges and corners was measured. Profilometry to measure the height was considered, but not performed due to the risk of equipment damage near the sample edges, as well as high risk of contaminating the probe with insufficiently dried photoresist.

### 3.2. Results on Edge Bead Reduction

The results are summarized in Table 1. It was found that chuck 1 produced samples with an edge bead with a width of 396 µm ± 42 µm, and corner width of 1181 µm ± 40 µm. The figure of 72% of the corners overhang outside the sample edge, and the smallest corner was 988 µm wide.

In contrast, chuck 2 produced statistically significantly smaller amounts of edge bead in the corners, at a significance level *p* = 0.05. The edges had a width of 339 µm ± 30 µm while width of the corner bead showed a very large uncertainty of 269 µm. This is the result of a bimodal distribution of corner widths, where 47% of the corners are larger than 1000 µm, and the remainder are smaller than 700 µm. This is mostly caused by the samples not being diced perfectly square and, therefore, not having all the corners in contact with the chuck. Only the corners that were in contact with the chuck exhibited the desired reduction in edge bead. These problems may be ameliorated by a chuck design with one or more movable edges. The resist along the edges was not significantly reduced.

**Figure 3:**
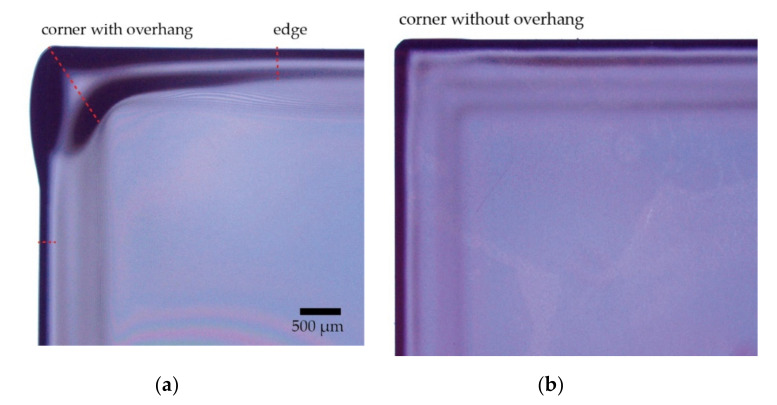
Samples spin-coated with OiR 908-35 resist at 2000 rpm for a target thickness of 5 µm. (**a**) Using a conventional chuck. A thick edge bead region is clearly visible. The measurements have been made along the red dashed lines. (**b**) Using the custom chuck. The edge bead is visibly reduced. The corner shown was in contact with the spinning chuck.

The full potential of the technique is shown in the row “Chuck 2 filtered” of Table 1, where only the corners that were in contact with the chuck were considered for the analysis. These data points show a 22% smaller width of the edge bead than obtained when using chuck 1 and 57% narrower corners, not accounting for uncertainty. It can also be clearly observed in Figure 3 that the overhang visible when using the original chuck disappears with the improved chuck.

Further improvements to the chuck are possible: resist stains on the bottom of the sample were observed, resulting from an imperfect vacuum seal. The addition of gutters to guide the leftover resist to the disk edge will reduce backside contamination and decrease the edge bead further.

## 4. Photolithography of Small Samples

The EV620 contact mask aligner used in this work is designed to work with 4-inch wafers. The mask alignment process is therefore not optimal for small samples. The mask contact step (i.e., wedge correction) carries the risk of crushing the sample. In this step, the mask may be brought down at an angle relative to the KY(WO_4_)_2_ surface, which concentrates the force on an edge or corner of the brittle KY(WO_4_)_2_ sample and may cause damage to both the sample and the mask. In the past, this was palliated by using cleanroom tissue as a compressive base together with surrounding the sample with glass pieces of matched thickness [28].

**Figure 4:**
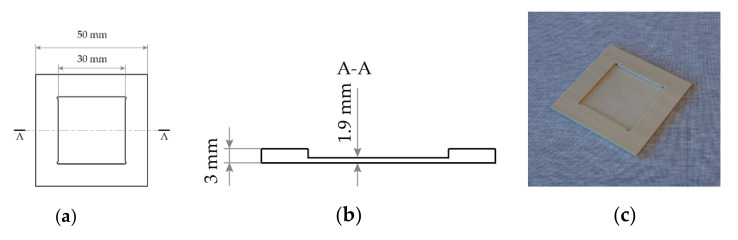
Technical drawing of the lithography sample mount. (**a**) Top-down view. (**b**) Cross-section through line A-A in (**a**), showing the geometry of the center of the mount. (**c**) Photograph of the lithography sample mount.

In this work, a sample mount is designed and manufactured, as shown schematically in Figure 4a,b and photographed in Figure 4c. The mount consists of a 3-mm thick square of width and length 50 mm × 50 mm, with a 1.9-mm deep blind hole of 30 mm × 30 mm in the middle. The mount is made from polyether ether ketone (PEEK) plastic due to its good properties for high-precision machining.

A pad of compressible material is placed inside the blind. In this work, 3 pieces of cleanroom tissue are used. The KY(WO_4_)_2_ sample is placed on top of the pad and the complete assembly is aligned to the mask by eye before mask contact is made. During contact, the KY(WO_4_)_2_ sample is gently pressed into the cleanroom tissue until flush with the top of the holder. The mask then rests on the large PEEK mount, which helps provide uniform pressure over the small KY(WO_4_)_2_ piece, especially when exposing dice far from the mask center. Although a quantitative analysis is challenging, we report that sample chipping has no longer been observed after this technique was implemented.

## 5. Deep Reactive Ion Etching

In inductively coupled plasma reactive ion etching (ICP-RIE), the general scheme is to ionize a gas and let reactive species chemically etch the sample in combination with physical sputtering. For materials like silicon and silicon oxide, ICP-RIE permits complex physical/chemical schemes with a wide parameter space to be used to tune the properties of the etched features [25,29,30,31]. However, as KY(WO_4_)_2_ is highly inert, this is not a viable option. The only volatile compound that is formed when using chlorine or fluorine chemistries is WF_6_, with an atmospheric pressure boiling point of 17.1 °C [32]. While an Ar/SF_6_ gas mixture has been reported for sample thinning [33], we found that the inclusion of SF_6_ at concentrations of ~10% reduces the selectivity with respect to photoresist from 2.6 to 0.3. For this reason, we opted to use in this work a purely argon-based etch, based entirely on sputtering and without chemical etch mechanisms.

**Figure 5:**
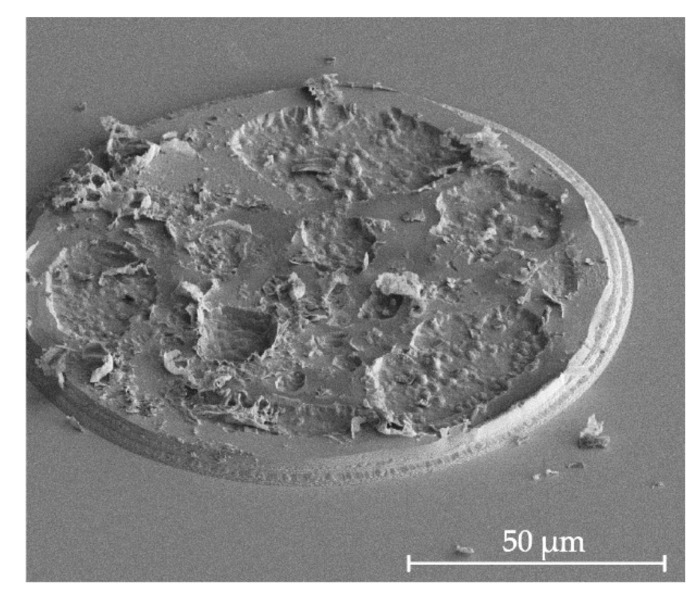
Severe reticulation of a 3.5-µm thick Olin OiR 908-35 photoresist used to pattern a 120-µm large disk.

One challenge of deep Ar etching is reticulation of the thick photoresist layer [26]. It is commonly observed that when the resist is etched for a long time by using argon, surface cracking, bubbling, and chemical hardening can occur. Such defects cause severe roughness, as well as craters in the sample where the resist has cracked, and they make resist removal challenging. An example is shown in Figure 5, where a disk of 120 µm in diameter in a 3.5-µm thick Olin OiR 908-35 photoresist was subjected to 30 min of argon plasma etching. Furthermore, the hardened resist may be challenging to remove using oxygen ashing or stripping in HNO_3_. While it may be assumed that hard-baking or UV curing, which are known to increase the chemical resistance of photoresist [34], would diminish the risk of reticulation, we observed the opposite effect in our experiments. Therefore, such hardening processes are not recommended for Ar etching.

To circumvent the issue of resist reticulation, hard masks such as SiO_2_, other oxides, or metals can be used, sometimes in combination with reactive species [27]. Some of the materials known to sputter slowly in Ar include photoresists, SiO_2_, amorphous carbon, and Al_2_O_3_ [35,36], which makes them candidates for use as hard masks. Chromium has also been used as a hard mask for KY(WO_4_)_2_ in a process incorporating SF_6_ [37], although this carries the risk of high optical losses due to metal contamination.

Some processes from this group and others using various mask materials are listed in Table 2. Further processes with SU-8 resists and SiO_2_ from the same experimenters have been reported. However, they are not documented here due to missing information on selectivity and performance. Geskus [38] and Sefunc [39] both reported high sidewall roughness in their respective processes, especially for deep etching, while Medina [37] did not comment on it.

Amorphous carbon offers extremely high selectivity, however, it is very fragile and requires plasma-enhanced chemical vapor deposition (PECVD) SiO_2_ adhesion and capping layers for protection. To avoid damaging the carbon layer during wet cleaning, it must be patterned using the SiO_2_ as a hard mask, which itself must be patterned using a second hard mask. This leads to a total of four depositions and four dry etching steps, with each carrying a risk of roughness transfer or damage.

### 5.1. Etching Procedure

A hard mask consisting of 5 µm thick SiO_2_ deposited by PECVD was utilized in this work. The SiO_2_ was patterned using a Olin OiR 907-17 photoresist. The resist was spin-coated at 4000 rpm, for a thickness of 1.7 µm. After contact photolithography using an EV620 mask aligner with a broadband source and the lithography tools described previously, the SiO_2_ hard mask layer was etched in an Adixen AMS100 DE ICP-RIE. The etching parameters are ICP power 2800 W, radiofrequency capacitively coupled plasma (RF CCP) power 350 W, C_4_F_8_ flow 20 sccm, CH_4_ flow 15 sccm, He flow 150 sccm, chamber pressure 6.4 mTorr, and table temperature −10 °C. This process has a selectivity of 5, which allows for a thinner photoresist layer than those mentioned previously in this work.

The KY(WO_4_)_2_ etching was performed in an Oxford PlasmaPro 100 Cobra. The etching parameters are ICP power 1875 W, RF CCP power 150 W, pressure 3 mTorr, Ar flow 90 sccm, and table temperature 10 °C. Using this recipe, a selectivity of 2.6 for KY(WO_4_)_2_ with respect to photoresist and 1.2 for KY(WO_4_)_2_ with respect to SiO_2_ was obtained. The KY(WO_4_)_2_ etch rate is 100 nm/minute.

The etching was performed for 32 min. After etching, any residual photoresist was removed using a TePla 300 O_2_ microwave asher. The structures were imaged using an FEI Nova focused ion beam/scanning electron microscope (FIB/ SEM).

### 5.2. Results on Dry Etching

**Figure 6:**
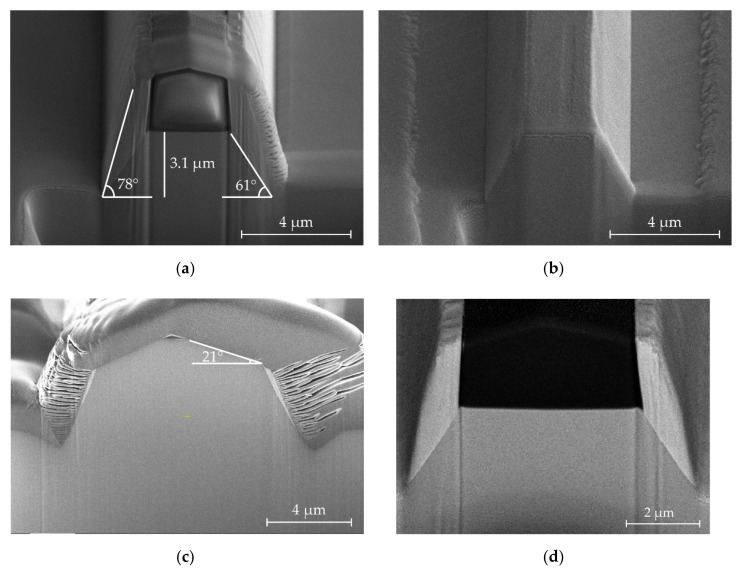
Focused ion beam (FIB) cross-sections of etched structures at various points during the processing. (**a**) A KY(WO_4_)_2_ immediately after dry etching. Significant redeposited ears extend above the structure, to the total height of the remaining SiO_2_ mask. The horizontal width of the redeposited material is 1 µm. A FIB-deposited Pt layer covers the cross-section to reduce geometric scanning electron microscope (SEM) artifacts and rounding during cross-sectioning. A sidewall angle of ~61° was measured for the KY(WO_4_)_2_ sidewall while the outer redeposition exhibits an angle of 78°. (**b**) A similar structure from the same batch after HCl etching and HF SiO_2_ hard mask stripping. (**c**) SEM image of a similar structure, etched to a depth of 6.5 µm. The SiO_2_ hard mask was completely consumed, which led to the second angle (21°) on the upper part of the sidewall. A thicker Pt layer has been used during the cross-sectioning. (**d**) FIB cross-section of a structure with significant redeposition imaged only using backscattered electrons. The redeposited material appears bright, indicating a higher concentration of heavy ions. In contrast, the SiO_2_ mask, which is composed of light atoms, appears very dark.

The resulting structure using a SiO_2_ hard mask and the etching recipe discussed above is shown in Figure 6a,b. The etch depth is 3.1 µm. The sidewall angle is 78°, while the measured angle of the structure within the redeposition is 61°.

Figure 6c shows a FIB cross-section of a larger structure etched almost to the point of completely removing the mask. The total etch depth is now 6.5 µm. The observed pentagonal structure results from SiO_2_ mask retraction because the mask etches more rapidly near the corners. At a given point during prolonged etching, the SiO_2_ mask is completely etched through at the edges of the patterns. Further etching at this point is possible, although a second slope appears as a result of transfer of the mask profile at the surface of the feature.

Furthermore, the reduced steepness of the sidewall decreases the amount of residual redeposition because of the higher received ion flux by the sidewall at that angle. The outer sidewall angle is now 67°, and the surface top has a 21° angle.

## 6. Redeposition-Free Structures in KY(WO_4_)_2_

Redeposition is easily avoided when etching materials such as Si and SiO_2_ by using inductively coupled plasma reactive ion etching (ICP-RIE) with fluorinated compounds like CHF_3_ and SF_6_, as the reactive F^-^ species form volatile compounds that are pumped away in gas phase [29]. When using only inert argon gas in ICP-RIE, however, the etching mechanism is purely physical and redeposition becomes a problem.

The three main strategies for masking prior to reactive ion etching are (i) conventional rectangular masks, (ii) rounded masks, and (iii) very thin hard masks [27]. However, all three approaches have advantages and disadvantages. A standard rectangular mask increases the amount of redeposition because the tall mask presents a large area for sputtered atoms to hit. This is clearly visible in Figure 6a. Additionally, the ion flux (i.e., the number of ions incident on the sidewalls) is low because the sidewall angle is steep, preventing efficient etching of the redeposition, which can build up over time. In ion beam etching (IBE), the standard solution is tilting and rotating the sample, which provides a more uniform ion flux. This is, however, not possible in ICP-RIE systems.

The second approach, the use of rounded masks, helps reduce the redeposition by presenting an angled sidewall. The ion flux at the slanted sidewall is higher because it is no longer parallel to the ion direction. The redeposition on the slanted sidewalls is therefore etched more rapidly, ideally to the point of total removal. A rounded mask is typically prepared by heating the resist to a temperature close to but below its melting point, so that it reflows. However, broadening of the resist patterns due to the reflow process causes a tradeoff in resolution. In addition, since the resist mask is thinner close to the edge, a deep etch will induce significant mask erosion, leading to rough, severely sloped sidewalls [39].

The third option is a thin hard mask. A thin mask does not provide a large wall for the redeposition to stick to, preventing the formation of the structures shown in Figure 6a. The material utilized as the hard mask should exhibit a significantly lower etch rate than the sample to be etched. Alternatively, the etch rate of the mask material should be considerably reduced by the addition of a chemical component into the gas mixture [27]. Past experiments have identified amorphous carbon as a candidate as thin hard mask material [40]. However, amorphous carbon is a very challenging material to work with due to fragility, low growth rate, and poor adhesion. Furthermore, simulations in Synopsys OptoDesigner Process Flow have shown that, although redeposition does not protrude vertically above the mask, the sidewalls remain sloped.

In this work, we present a novel fourth approach, which consists of a chemical cleaning step following the ICP-RIE dry etch with a square mask. Even though KY(WO_4_)_2_ is a chemically inert material, there are references to chemical reactions occurring at elevated temperatures [41]:

(1) $$\mathrm{KY}{\left({\mathrm{WO}}_{4}\right)}_{2}+4\;\mathrm{HCl}\overset{100\;{}^{\circ}C}{\to }\;\mathrm{KCl}+{\mathrm{YCl}}_{3}+2{\mathrm{WO}}_{3}+2{\;H}_{2}O$$

In previous work, this reaction has been used to selectively under-etch amorphized KY(WO_4_)_2_ [20], followed by a tetramethylammonium hydroxide (TMAH) etch to remove the insoluble by-product WO_3_. Here, the procedure is performed using a concentration of 20% HCl and a temperature of 80 °C for 15 min to remove the redeposition attached to the sidewalls of the deeply etched KY(WO_4_)_2_ structures. The concentration and temperature were designed to minimize the risk of harmful HCl fumes while maximizing effectiveness.

To evaluate the etch rate of crystalline KY(WO_4_)_2_ in a 20% HCl solution at 80°C, a KY(WO_4_)_2_ sample with a patterned plasma enhanced chemical vapor deposition (PECVD) SiO_2_ mask was immersed in the acid solution for 2 h. The resulting step height after wet etching was measured to be 120 nm ± 5 nm, yielding an etch rate of 1 nm/minute. This rate is low enough that the acid can safely be used on KY(WO_4_)_2_ structures. However, the roughness of the surface of the KY(WO_4_)_2_ increases from <1 nm to ±5 nm. To prevent this increase in roughness, the SiO_2_ mask should not be removed prior to redeposition stripping.

### Results of Redeposition Removal

Figure 6a,b showa a 3.1-µm deep etched structure immediately after dry etching (Figure 6a) and after the stripping of the redeposition by the proposed wet etching step (Figure 6b). The redeposition produced by dry etching is significant, extending upward as high as the residual hard mask and up to 1 µm laterally. The lateral growth of the redeposition increases the effective width of the mask during the etching process, decreasing the angle of the KY(WO_4_)_2_ sidewall to ~61°. In contrast, the sidewall of the redeposition is more vertical at 78°. In Figure 6b, the redeposition has been removed using the proposed wet etching step, with the structure showing a distinctly pyramidal shape, with smooth sidewalls.

Figure 6d shows a similar structure, imaged with an Everhart–Thornley detector with a negative bias voltage. This configuration repels secondary electrons and detects only backscattered electrons. The backscattered electron yield is strongly dependent on elemental composition, with heavier atoms having a higher yield and appearing brighter [42,43]. The redeposition is much brighter than the crystalline KY(WO_4_)_2_. This indicates that it is not simply an amorphous phase of KY(WO_4_)_2_, but rather a stoichiometrically different compound.

It is also known that heavier atoms sputter omnidirectionally, while lighter elements to a greater extent sputter normal to the surface of the sample [44]. Oxygen, potassium, yttrium, and tungsten have atomic numbers 8, 19, 39, and 74. It is therefore likely that the redeposition is rich in metals, especially tungsten, as these would have been sputtered onto the features of the sidewalls and appear brighter.

The exact composition of the redeposited material is not known. Techniques like energy-dispersive X-ray spectroscopy (EDX) and X-ray photoelectron spectroscopy (XPS) may be used, however, the redeposited structures are very small. It is therefore challenging to get high quantitative accuracy without measuring the substrate. The etching mechanism is also not known. The etching of KY(WO_4_)_2_ relies on dissolving the salt into its constituent complex ions, and this is not transferable to HCl reacting with a partially oxidized alloy.

**Figure 7:**
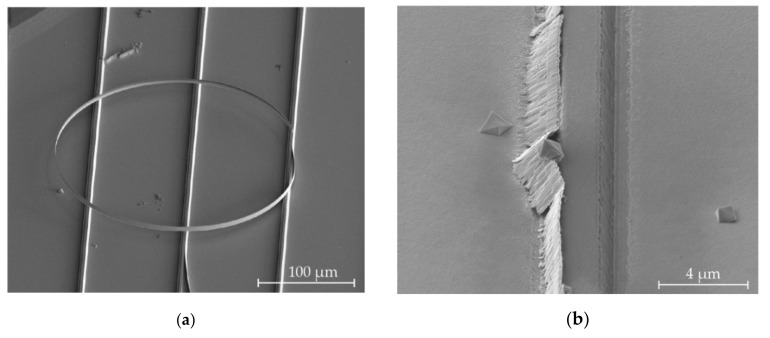

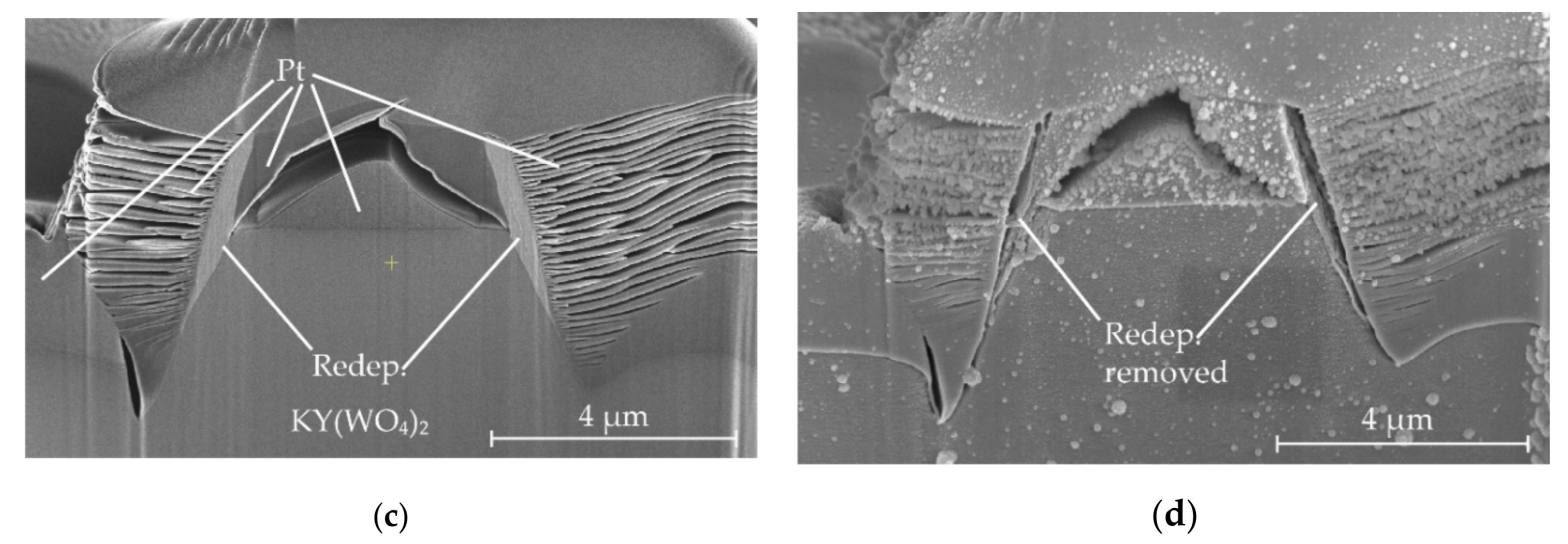
(**a**) A strip of sidewall redeposition that has delaminated during ultrasound cleaning and curled up due to internal shear stresses. (**b**) Sidewall redeposition that has been partially removed during an insufficiently long HCl etching step. (**c**) Cross-section of a structure with significant redeposition, covered with a thick layer of platinum. (**d**) The same cross-section after a 5 min HCl etching step. Most of the redeposited material is removed.

Figure 7 shows redeposition peeling away after ultrasound cleaning (Figure 7a) and after a 5 min long etch step (Figure 7b), which is insufficient time for complete removal. This poses the question of whether the removal mechanism is etching or peeling away from the KY(WO_4_)_2_ sidewalls following intrusion by the etchant. Such intrusion may happen from the bottom, where argon ion bombardment may have amorphized a thin KY(WO_4_)_2_ layer and made it susceptible to etching.

However, Figure 7c shows the cross-section of a structure with significant redeposition, covered with a thick layer of FIB-deposited platinum. Figure 7d shows the same cross-section after the same incomplete etching step that produced the structure in Figure 7b. The redeposition is clearly reduced, with complete removal on the right and only a thin sliver remaining on the left. This demonstrates that redeposited material is eroded by the etch and does not simply delaminate and float away in one piece. The stripping mechanism is therefore likely a combination of delamination and etching.

**Figure 8:**
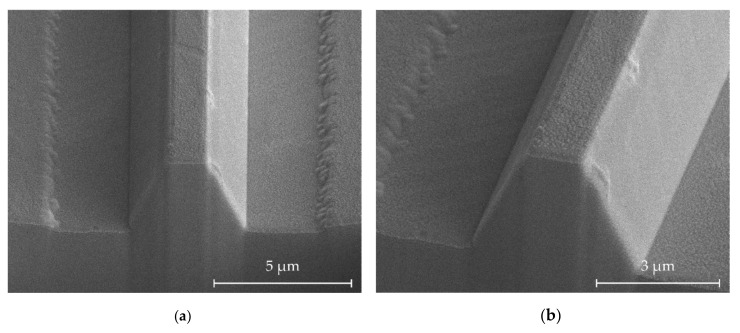
Ridge structures after redeposition stripping. The feature width at the top is 1.4 µm, the etch depth is 3.1 µm, the width at the bottom is 4.2 µm, and the sidewall angle is 67°. (**a**) Normal view, (**b**) 15° rotated view of the same structure, showing low sidewall roughness.

Figure 8 shows an example of the highest-achieved resolution deep etched structure with smooth sidewalls. The width at the top is 1.4 µm and the depth is 3.1 µm, with an angle of 67°. As has been shown previously, deeper structures are achievable. However, the risk of sidewall roughness due to cumulative micromasking increases over time. Narrower structures may also be made, however, the main contribution to feature size is the sidewall slope.

## 7. Conclusions

Several techniques have been introduced that minimize or eliminate common challenges in KY(WO_4_)_2_ fabrication for high aspect ratio structures. Together, these techniques improve the performance and yield of the KY(WO_4_)_2_ lithography and etching process. The useful processing area of small samples has been increased through edge bead reduction. The yield of the UV contact lithography process step has been increased to 100%. A deep etching procedure has been developed, which, in combination with a technique for removing redeposition, has been demonstrated to produce deep (3.1 µm) KY(WO_4_)_2_ structures with clean smooth sidewalls with an angle of 67°. Very deep (6.5 µm) features with a pentagonal structure due to full consumption of the SiO_2_ hard mask have also been demonstrated. The microfabrication processes described here are directly applicable to existing KY(WO_4_)_2_ processes such as thin layer lapping and polishing or ion irradiation to fabricate high refractive index contrast waveguides and other devices. The techniques may also be applicable to other hard-to-process materials.

## Figures and Tables

**Table 1:** Summary of the edge bead measurements, showing the edge bead width, corner bead width, percentage of corner beads overhanging the chip corner, and percentage of corner beads larger than 1 mm. Edge and corner lengths are given as the average and 95% error bound calculated using a Student’s T distribution. Four samples were used to test each of the chucks.

	Edge (µm)	Corner (µm)	Overhang (%)	>1 mm (%)
Chuck 1	396 ± 42	1181 ± 40	72	95
Chuck 2	339 ± 30	925 ± 269	18	47
Chuck 2 filtered	310 ± 34	508 ± 82	14	0

**Table 2:** Overview of ICP-RIE etching processes used in this work and in previous works, using OiR series photoresists and various hard mask materials.

	Martinussen et al.			Sefunc [39]	Geskus [38]	Medina [37]
Machine	Oxford PlasmaPro 100 Cobra			Adixen AMS100DE	Oxford Plasmalab 100	Oxford PlasmaPro NGP80
Mask	OiR	SiO_2_	C [40]	OiR	Al_2_O_3_	Cr
ICP power [W]	1875	1875	1875	1500	1500	350
CCP power [W]	150	150	150	150	150	210
Ar %	100%	100%	100%	100%	100%	50%
SF 6%	-	-	-	-	-	50%
Pressure [mTorr]	3	3	3	3.75	19	12
Etch rate [nm/min]	100	100	100	65	84	73
Selectivity	2.6	1.2	8.2	1.2	2.7	5
